# Targeting the Complement Pathway as a Therapeutic Strategy in Lung Cancer

**DOI:** 10.3389/fimmu.2019.00954

**Published:** 2019-05-10

**Authors:** Emily K. Kleczko, Jeff W. Kwak, Erin L. Schenk, Raphael A. Nemenoff

**Affiliations:** Department of Medicine, University of Colorado Anschutz Medical Campus, Aurora, CO, United States

**Keywords:** lung cancer, complement-immunological terms, oncogene, immunotherapy, microenvironment

## Abstract

Lung cancer is the leading cause of cancer death in men and women. Lung adenocarcinoma (LUAD), represents approximately 40% of all lung cancer cases. Advances in recent years, such as the identification of oncogenes and the use of immunotherapies, have changed the treatment of LUAD. Yet survival rates still remain low. Additionally, there is still a gap in understanding the molecular and cellular interactions between cancer cells and the immune tumor microenvironment (TME). Defining how cancer cells with distinct oncogenic drivers interact with the TME and new strategies for enhancing anti-tumor immunity are greatly needed. The complement cascade, a central part of the innate immune system, plays an important role in regulation of adaptive immunity. Initially it was proposed that complement activation on the surface of cancer cells would inhibit cancer progression via membrane attack complex (MAC)-dependent killing. However, data from several groups have shown that complement activation promotes cancer progression, probably through the actions of anaphylatoxins (C3a and C5a) on the TME and engagement of immunoevasive pathways. While originally shown to be produced in the liver, recent studies show localized complement production in numerous cell types including immune cells and tumor cells. These results suggest that complement inhibitory drugs may represent a powerful new approach for treatment of NSCLC, and numerous new anti-complement drugs are in clinical development. However, the mechanisms by which complement is activated and affects tumor progression are not well understood. Furthermore, the role of local complement production vs. systemic activation has not been carefully examined. This review will focus on our current understanding of complement action in LUAD, and describe gaps in our knowledge critical for advancing complement therapy into the clinic.

## Introduction

**Figure 1:**
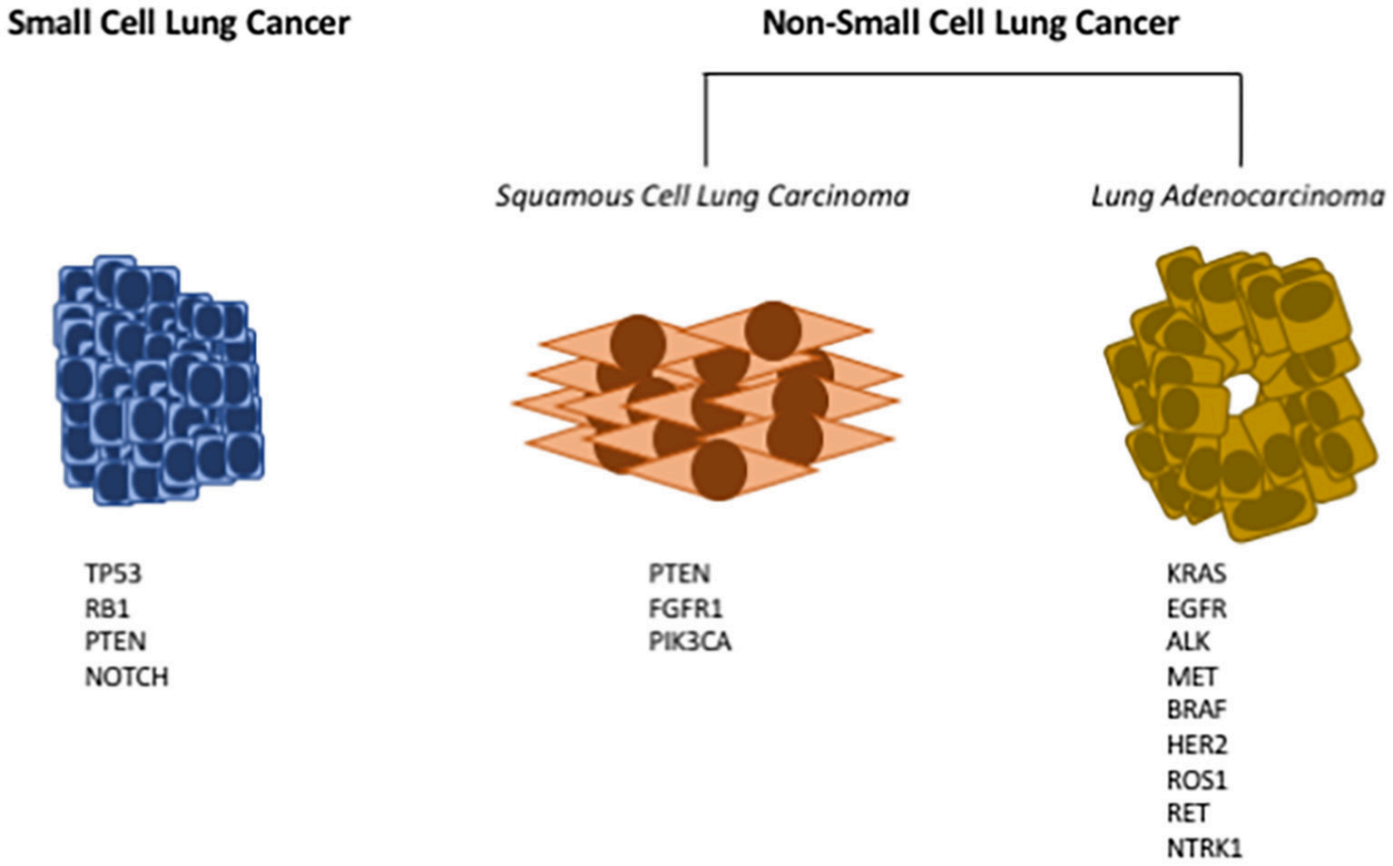
Common driver mutations in lung cancer. Lung cancers have historically been subdivided into either small cell lung cancer (SCLC), or non-small cell lung cancer (NSCLC). Small cell lung cancer has few oncogenic driver mutations; here are listed the most frequently identified genetic mutations in SCLC **(Left)**. NSCLC can further be subdivided into squamous cell lung carcinoma (SCC), or lung adenocarcinoma (LUAD). Multiple oncogenic drivers have been identified in LUAD **(Right)**; for many of these targeted therapies have been developed. For SCC **(Middle)**, there are fewer identified oncogenic drivers, and no targeted therapies have been approved.

Lung cancer is the leading cause of cancer death in both men and women (1). While there is clearly an established risk for lung cancer associated with cigarette smoking, recent data indicate an increased risk of lung cancer in never smokers, especially in women (1). Thus, while decreased rates of cigarette smoking should lower the incidence of lung cancer, lung cancer will remain a major cause of cancer death. In spite of active research identifying new therapeutic targets, the overall survival rate for lung cancer still remains discouragingly low, underscoring the need for both new preventive and therapeutic approaches. Historically, lung cancer has been subdivided based on histology into two major subtypes: non-small cell lung cancer and small cell lung cancer (see Figure 1). About 85% of lung cancers are non-small cell lung cancer (NSCLC) with small cell lung cancer (SCLC) making up the majority of the remainder. There are a few other minor types of lung cancer such as large cell carcinoma, adenosquamous cell carcinoma, and sarcomatoid carcinoma, but these are rare. SCLC typically express a range of neuroendocrine markers and transcription factors that play crucial roles in their differentiation (2, 3).

NSCLC has further been subdivided into adenocarcinoma and squamous cell carcinoma (see Figure 1). These classifications are based on cells of origin as well as histology. Squamous cell lung cancer (SCC) generally arises from the proximal airway while adenocarcinomas develop from more distal locations (4). SCC begins in the squamous cells that make up the alveolar-capillary membrane, the only barrier between the air in the lungs and the capillary blood. Tracheal basal cell progenitors have been speculated to be the origin in mouse lung SCC due to the fact that the gene expression and histopathology patterns of SCC frequently resemble these cells (5, 6). About 30% of all lung cancers are classified as squamous cell lung cancer. It is more strongly associated with smoking than any other type of NSCLC. While numerous oncogenic drivers have been identified for lung adenocarcinoma, it has been more challenging to identify drivers for SCC (7, 8). Adenocarcinoma (Greek: adenos, gland plus karkinos, cancer) is a cancer that begins in cells in the glands. Using genetic models it has been demonstrated that lung adenocarcinomas (LUAD) originate from either type II pneumocytes or Clara cells (9). In addition, earlier studies have identified a bronchioalveolar stem cell population as being the potential cell of origin (10). Adenocarcinoma accounts for approximately 40% of all lung cancers.

Two major advances have occurred during the past decade which hold promise for the treatment of lung cancer, particularly LUAD. The first of these is the identification of multiple oncogenic drivers and the recognition that subdividing LUAD based on these drivers will dictate therapy. This has resulted in the development of multiple targeted therapies which have been approved for treatment of subsets of LUAD. The second breakthrough is the advent of novel immunotherapy approaches, specifically the use of antibodies targeting immune checkpoint inhibitors. These have been shown to be effective in NSCLC and are approved for subsets of LUAD as well as for SCC (11–14). Nevertheless, in spite of these novel approaches, the overall survival rate for NSCLC has not significantly improved, underscoring the need for new therapeutic approaches. As discussed below, therapeutic approaches are particularly constrained by the oncogenic drivers. There are currently no approved agents targeting K-Ras dependent lung cancer; however, this subset of patients show a response to immune checkpoint inhibitors (15). In contrast, while numerous targeted therapies are approved for LUAD driven by mutations in tyrosine kinase receptors, these patients show a very poor response rate to immune checkpoint inhibitors (16). In going forward, it is therefore critical to integrate our preclinical knowledge to define how specific oncogenes engage the immune tumor microenvironment. This review will focus on the complement pathway, largely in LUAD. Once considered a pathway associated with inhibition of tumor initiation and progression, it has become clear from work of multiple groups that complement is in fact complex and can actually promote progression of multiple cancers, including LUAD through promoting inflammation and regulating immunosuppressive pathways. These studies suggest that targeting complement either as monotherapy, or in combination with other immunotherapies represents a novel strategy for treatment, and possibly prevention of lung cancer.

## Oncogenic Drivers and Targeted Therapies

Studies performed during the past 15 years have subdivided lung adenocarcinoma according to the dominant oncogenic driver (17, 18). This has resulted in a paradigm shift in the treatment of this disease. Whereas, earlier clinical studies had tested potential therapeutic agents in an unselected group of patients, discovery of distinct oncogenic drivers has resulted in targeted therapies against that dominant oncogene, with the concept of patient selection becoming standard of care. This has led to a focus on criteria for patient selection, with the model being to seek strong responses in a subset of patients rather than a more modest response across all patients. LUAD can be defined at the molecular level by recurrent “driver” mutations or amplifications, including, but not limited to: *ALK, BRAF, EGFR, FGFR1, KRAS, MET, RET, NTRK1*, and *ROS1*. These have been extensively reviewed elsewhere (18). The National Comprehensive Cancer Network (NCCN) guidelines now recommend routine testing for *NTRK, ALK, ROS1, BRAF*, and *EGFR* for all new cases of advanced lung adenocarcinoma for which we have therapies. Currently, personalized therapies that identify and target specific biomarkers have resulted in substantial benefits for NSCLC patients with *EGFR* mutations, gene alterations involving the anaplastic lymphoma kinase (*ALK*) gene, *BRAF* V600E mutation, or the *ROS1* gene. The common genomic alterations, frequencies, and current FDA-approved therapies to target the known mutations in NSCLC are summarized in Table 1 (19). In this review we will briefly discuss EGFR, ALK, and K-Ras gene alterations in lung adenocarcinoma.

**Table 1:** Genomic alterations of lung cancer.

	**Type of alteration**	**Frequency (%)**	**FDA approved therapy**
EGFR	Mutation	10–35	Yes
KRAS	Mutation	25–30	No
FGFR-1	Amplification	20	No
ALK	Rearrangement	5–7	Yes
MET	Amplification	2–4	Yes, but for a different mutation
ROS1	Rearrangement	1	Yes, but for a different mutation
RET	Rearrangement	1	Yes, but only for other cancers
BRAF	Mutation	1–3	Yes

*EGFR, epidermal growth factor receptor; KRAS, Kristen RAt Sarcoma; FGFR-1, fibroblast growth factor receptor-1; ALK, anaplastic lymphoma kinase; MET, hepatocyte growth factor receptor. ALK, MET, ROS1, and RET are proto-oncogenes that arise from chromosomal rearrangements that generate a fusion gene, resulting in the constitutive activation of kinase domain*.

The epidermal growth factor receptor (*EGFR*) belongs to the avian erythroblastic leukemia viral oncogene homolog (*ERBB*) family, or also known as the *Her* family, that includes 4 different receptors: EGFR, ErbB2, ErbB3, and ErbB4 (20). EGFR is overexpressed in many cancers, including NSCLC, and several somatic mutations have been detected in NSCLC. The most prevalent mutation in the EGFR kinase domain—accounting for approximately 45%—is the inframe deletion of exon 19 between residues 747–750 (21). Another recurrent mutation that compromises another 45% of EGFR mutations is the mutation in exon 21 at the position 858 of kinase domain from a leucine (L) to an arginine (R). Exon 18 substitution and exon 20 in-frame insertions account for the rest. These gain-of-function EGFR mutations lead to constitutive phosphorylation and activation of cell survival and proliferation pathways (22). Targeting the EGFR with “first-generation” tyrosine kinase inhibitors (TKIs) such as gefitinib and erlotinib has been approved since 2003 for NSCLC. These TKIs compete with ATP in a reversible manner to bind the kinase domain of the receptor. Although initial responses in patients to these TKI agents can be dramatic, most patients will eventually relapse due to the acquisition of drug resistance, a common observation among many targeted therapies. Multiple mechanisms of acquired resistance to targeted EGFR therapy have been discovered in patients. Patients who became resistant to first generation EGFR TKIs often acquire a T790M somatic mutation, which has been designated a “gatekeeper” mutation (23) that increases affinity for ATP (24). Additional resistance mechanisms include amplification of hepatocyte growth factor receptor (*MET*), observed in 5–15% of patients who received first generation EGFR TKIs (25, 26). MET signaling activates MAPK and PI3K/AKT pathways, bypassing the requirement for EGFR signaling. To address the problem of the multiple mechanisms of resistance, second and third generation EGFR inhibitors have been developed. The defining characteristic of the third-generation EGFR TKIs is that they have significantly greater activity against EGFR mutant receptors than EGFR wildtype (WT), making them more sensitive for tumor cells (27). Osimertinib, a third-generation EGFR TKI, has shown objective response rates and progression-free survival compared with chemotherapy in the first-line setting and was recently approved as the first-line treatment for *EGFR*-mutated NSCLC (28). Despite its success, there are reports of an acquired mutation at C797S in exon 20 among the patients who received osimertinib which affects drug binding, rendering the TKI ineffective (29). Acquired resistance through activation of Aurora A kinase has also been reported (30).

Anaplastic Lymphoma Kinase (ALK) is a receptor tyrosine kinase that in normal settings signals to promote cell growth and inhibit apoptosis in a regulated manner. Rearrangement of the *ALK* gene results in the N-terminal fusion of the ALK tyrosine kinase domain with different fusion partners, mainly echinoderm microtubule-associated protein like 4 (*EML4*), producing EML4-ALK fusion proteins. In other cases, ALK is shown to also partner with kinesin family member 5B (*KIF5B*) (31) or TRK-fused gene (*TFG*) (32). The dimerization of ALK mediated by its fusion partner results in a constitutive activation of the ALK tyrosine kinase activity and subsequently mediates an increase in pro-growth and anti-apoptotic signaling in NSCLC (33, 34). Similarly, there are described other fusion proteins resulting from the chromosomal rearrangement such as *RET* fusion with *KIF5B* (35), *ROS1* fusion with *CD74* (36), *NTRK1* fusion with myosin phosphatase Rho-interacting protein gene (*MPRIP*) or *CD74* (37). Approximately 5–7% of NSCLC patients harbor ALK fusions (38). In an initial Phase I trial, the patients with *ALK* rearrangements displayed a 60.8% objective response rate to the ALK/ROS1/MET TKI, crizotinib (39). The median progression-free survival (PFS) was 9.7 months with the probability of PFS at 6 months to be 87.9%. The second-generation ALK-inhibitor ceritinib also showed a 60% response rate among the 180 ALK-fusion positive NSCLC patients in a phase I trial (40). An *EGFR* L858R mutation, *ALK* gene amplification, *KRAS* mutation, and *KIT* gene amplification have been reported in ALK fusion positive patients with acquired resistance to crizotinib, suggesting that other genetic changes may confer crizotinib resistance. Novel therapeutic strategies to overcome the development of acquired resistance to ALK TKIs are currently being studied (18).

Kristen Rat Sarcoma (KRAS) is a small GTPase that is activated when a GTP is bound and deactivated when KRAS hydrolyzes GTP into GDP. Activation is mediated by the exchange of GDP to GTP and is facilitated by Guanine Nucleotide Exchange Factors (GEFs), whereas the deactivation mechanism of promoting GTP-GDP hydrolysis is mediated by GTPase Activating Protein (GAP). KRAS is a central protein that couples growth factor receptor signaling to downstream pathways including RAF-MEK-ERK and PI3K-AKT and is critical for cell proliferation and survival (41). Somatic mutations in KRAS are common in NSCLC occurring in ~15–25% of NSCLC patients. Common mutations are in amino acid residues 12, 61, and rarely on 13. These mutations will block GAP leading to constitutive activation of RAS. In lung adenocarcinoma, the common G12C mutation is a distinct feature of exposure to tobacco smoke. In spite of intense research, there are currently no agents to directly target KRAS (42). Therefore, many have elected to target pathways downstream of RAS, especially the RAF-MEK-ERK and PI3K-AKT pathways (43).

The findings summarized above have changed the way that lung cancer patients are treated. The standard of care upon diagnosis today is to perform genetic analysis to identify driver mutations. Patients with targetable drivers are placed on specific agents, and in general show an initial response characterized by tumor shrinkage. However, since resistance eventually develops in these patients, and there is a large fraction of LUAD patients where the oncogenic driver cannot be targeted (e.g., K-Ras) or in which there is no identifiable driver, additional therapeutic approaches are required. The relationship between specific oncogenic drivers and engagement of the complement pathway has not been established. However, as discussed below, specific oncogenic drivers in LUAD are associated with different sensitivity to immunotherapy, and thus complement activation needs to be studied in the context of specific oncogenes.

## Responses to Immunotherapy

A second major advance in the treatment of lung cancer has been the advent of immunotherapy. While lung cancer was thought for many years to not be an immunological cancer, recent studies have clearly demonstrated the contrary. In fact, lung adenocarcinoma as a subtype is one of the most immunological tumor types, and immunotherapy has been actively investigated in both NSCLC and SCC (13, 44, 45). Cancer cells can be recognized by the immune system due to their ability to express altered levels of cellular proteins or the expression of mutated proteins. However, tumors are rarely eliminated by activated T cells. A model to account for this has been proposed and designated “immunoediting” (46). In this model there is initial recognition of cancer cells by the adaptive immune system; however, eventually cancer cells adapt by engaging immunosuppressive pathways to counter T cell-mediated tumor killing. In fact, immunoevasion has been designated as one of the “Hallmarks of Cancer” (47). Targeting immunosuppressive pathways will presumably lead to reactivation of cytotoxic T cells and tumor elimination (13, 48, 49).

A great deal of research has focused on pathways that regulate the function of T cells under non-cancerous conditions, designated immune checkpoints (50, 51). These pathways function through specific ligand-receptor interactions to inhibit T cell function (52). The PD-L1 pathway involves expression of PD-1 on activated T cells (both CD8^+^ and CD4^+^), and PD-L1 which is expressed on cancer cells as well as inflammatory cells of the tumor microenvironment including macrophages. Binding of PD-L1 to PD-1 results in inhibition of T cell receptor signaling and generates an “exhausted” phenotype, thereby allowing tumor progression. Monoclonal antibodies that block these interactions result in reactivation of T cells, and potentially tumor elimination. For lung cancer, monoclonal antibodies against both PD-1 and PD-L1 have shown clinical efficacy, leading to their approval by the FDA (53). To date immune checkpoint inhibitors targeting the PD-1/PD-L1 pathway have been approved for lung adenocarcinoma. However, the overall response rate in unselected patients is approximately 20% (45), underscoring the need for additional therapeutic approaches. Even more discouraging are the data examining LUAD with driver mutations in tyrosine kinases (e.g., EGFR and ALK fusions). For this subgroup of patients the response rates are even worse, and for ALK fusions there are very few reports of a positive response to anti-PD-1/PD-L1 therapy (16). Current clinical trials are examining combinations of these agents, such as EGFR inhibitors and anti-PD-1 (54). There are a large number of factors that have been shown to correlate with responsiveness to checkpoint inhibitors. While correlations of mutational burden, the presence of neoantigens (55), cigarette smoking, and expression of PD-L1 have been associated with clinical response (45, 56), the cellular and molecular mechanisms mediating response to immune checkpoint inhibitors are not well understood. There is a concerted effort to combine checkpoint inhibitors with other agents, resulting in a large number of clinical trials, many with limited scientific rationale (57). To develop a more rational approach, a better understanding of the immune response in lung cancer is required. This will entail a more comprehensive examination of the changes in the tumor microenvironment, focusing on both the innate and the adaptive immune response and the cross talk between these pathways. In particular, for LUAD, it will be critical to integrate how specific oncogenic drivers regulate this interaction.

## Complement Pathway

**Figure 2:**
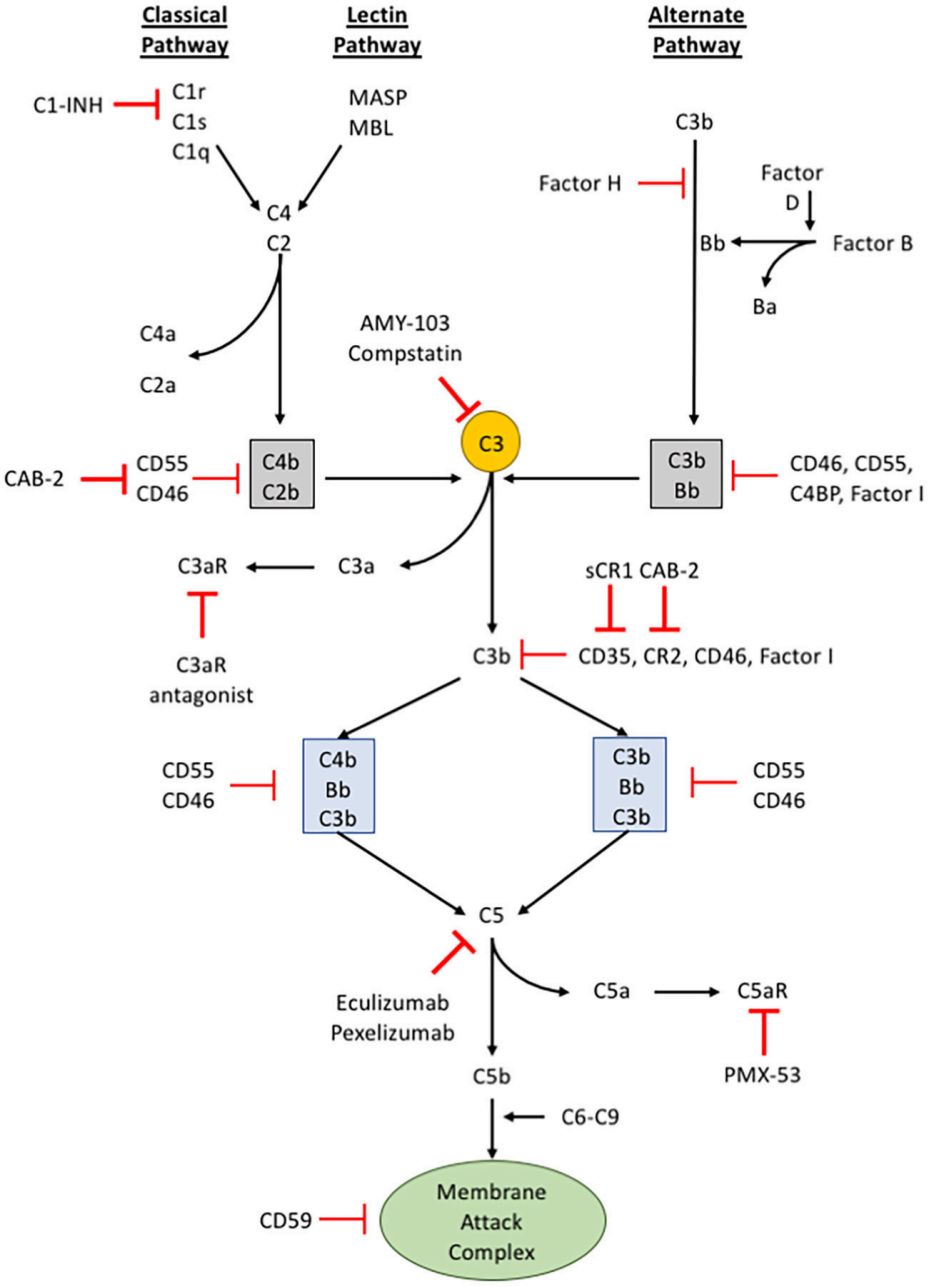
The complement pathway. A schematic of the complement signaling pathway where all 3 pathways (classical, lectin, and alternative) converge on C3. The red, bolded inhibitory signs indicate points in the complement signaling cascade where pharmacologic inhibitors can be used to alter signaling within cells, while the red, unbolded inhibitory sign indicates regulatory proteins in the complement cascade.

The complement pathway is part of the innate immune system that complements the ability of immunoglobulins and phagocytic cells to clear microbes and damaged cells, promotes inflammation through recruiting both the innate and adaptive immune cells, and attacks the pathogen's cell membrane itself. The complement pathway has been extensively reviewed (58–61), and therefore we will briefly discuss aspects of this pathway relevant to cancer progression. Many of the proteins that are involved in the complement pathway are synthesized by the liver and circulate as inactive precursors, or pro-proteins (see Figure 2). When stimulated, proteases in the system cleave the complement proteins in an amplifying cascade to release cytokines while complement fixation tags the triggering cells for opsonization. On the surface, the complement pathway may appear to be merely an antimicrobial mediator, but in the past few decades it has become apparent that such an intricate system has the potential to recognize surface antigens and may have much broader functions in immune surveillance and homeostasis (61). Furthermore, more recent studies have broadened the reach of complement activation from just the confines of intravascular systems, to local secretion of complement components by tissue and infiltrating cells, and potentially even intracellular activation of complement (62). Due to such broad targets and even greater functional versatility, the complement system is under tight regulation through multiple mechanisms (63, 64).

The activation of complement component 3 (C3) may occur through three distinct pathways: (i) classical pathway, (ii) alternative pathway, and (iii) lectin pathway (65) (see Figure 2). Although different proteins are recognized and involved, these different pathways of activation converge upon a single event, the conversion of C3 into C3a and C3b. The classical pathway begins when circulating immunoglobulins such as IgM or certain subclasses of IgG first bind to the antigen on the surface of the pathogen or target cells. This mediates the recruitment and activation of C1 complex comprising C1q, C1r, and C1s which is a serine protease that will subsequently cleave C2 and C4. The activated products of C2 and C4 form C4b2b, an assembly of multiprotein complexes with enzymatic activity termed, C3 “convertases.” The alternative pathway involves two distinct and separate initiation steps: (ii-a) properidin-mediated or (ii-b) C3(H_2_O)-mediated activation of C3. In properidin-mediated alternative pathway, properidin binds to C3b and activates Factor B and Factor D to form C3bBb, another C3-convertase. On the other hand, C3(H_2_O)-mediated activation involves the direct activation of C3 by Factor B and C3(H_2_O). C3(H_2_O) is the hydrolytic and conformationally rearranged product of C3 that functionally mimics C3b. This pathway results in C3(H_2_O)Bb, another C3-convertase. Lastly, the lectin pathway is triggered by carbohydrates recognized by mannose-binding lectin (MBL), ficolins, or collectin-11 which activates serine proteases such as MASP-1 and MASP-2. MASP-1 and MASP-2 are responsible for cleaving C2 and C4 to form a C3-convertase analogous to the C3-convertase made by the classical pathway. None of these pathways are exclusive in any disease and may occur simultaneously.

The activation of C3 produces C3a and C3b. C3a is a potent anaphylatoxin that promotes inflammation, cell migration, and activation. C3b, on the other hand, binds covalently to the surface of target cells through a newly exposed thioester bond and aids in opsonization (a process that increases the efficacy of the phagocytic process) or recruits other proteins with proteolytic properties to continue the complement activation cascade. C3b can bind to an existing C3 convertase such as C4b2b or C3bBb to form a C5 convertase, C4b2b^*^C3b or C3bBb^*^C3b, respectively. Similar to C3, cleaving C5 leads to production of the anaphylatoxin, C5a, and C5b. C5b recruits C6-9 to form the membrane attack complex (MAC) that causes pore formation and eventually cell lysis. The C3b-mediated opsonization or formation of MAC are thought to be the two main direct mechanisms of complement-mediated innate immune response (60, 61, 66).

It was originally thought that activation of complement would represent a strategy to inhibit tumor formation and progression, specifically through antibody mediated killing of tumor cells. Consistent with this model, in a genetic mouse model of breast cancer, autochthonous mammary carcinoma formation is accelerated in mice with global deletion of complement C3 (67). This is associated with alterations in the TME, promoting a more immunosuppressive environment. In particular, increases in regulatory T cells (Tregs) are observed in the setting of C3 loss. This is consistent with other studies demonstrating that anaphylatoxins regulate the development and recruitment of Tregs (68, 69).

However, several research groups have shown that complement deficiency or therapeutic complement inhibitors slow tumor growth in animal models (70–75). Published data also shows that the complement system is activated in many human patients with lung cancer (72, 76). Furthermore, examination of data in the Cancer Genome Atlas (TCGA) reveals gene amplification or increased expression of the complement regulatory proteins, CD46 and CD55, in approximately 25% of human lung adenocarcinomas. This suggests that tumors evolve the ability to block complement activation. These findings present a paradox: complement activation can promote tumor growth, yet cancer cells overexpress proteins that limit complement activation.

## Role of Anaphylatoxins

Activation of C3 and C5 generates C3a and C5a, respectively, and these anaphylatoxins are potent pro-inflammatory molecules that induce a multitude of effects on cells such as attracting neutrophils and monocytes to the site of complement activation. C3a and C5a sustains the inflammatory responses by activating granulocytes and macrophages, increasing vasodilation and vascular permeability and releasing pro-inflammatory mediators (61, 77). C3a and C5a exert their effects by signaling to their respective receptors, namely C3a receptor (C3aR), C5a receptor (C5aR) and C5a receptor-like 2 (C5L2). Both C3aR and C5aR belong to a family of transmembrane G protein coupled receptors, but C5L2 is not coupled to G proteins (78, 79). C5L2 was first described by Ohno et al., though its exact biological effects of signaling remain unclear (80). Many different cells express receptors for C3a and C5a. These include cells of myeloid origin (81), non-myeloid origin (82), dendritic cells (83), monocyte/macrophages (84), and neutrophils (85).

The host immune response has major effects on cancer initiation, progression, and metastasis (86). Since C3aR and C5aR are expressed on multiple immune cells, it has been postulated that an important function of complement is to regulate immunomodulatory functions of the tumor microenvironment. However, the role of anaphylatoxins in cancer progression is likely to vary between types of cancer and be context dependent. C5a stimulation has been shown to increase the release of matrix metalloproteinases (MMP) *in vitro*, while C5a-C5aR signaling enhances invasion of a human cholagiocellular carcinoma cell line (HuCCT1) *in vivo* (87). On a similar note, C5a-overexpressing lymphoma cells significantly accelerated tumor progression. The authors attributed this to increased recruitment of Gr-1^+^CD11b^+^ myeloid cells in the spleen and overall decreased CD4^+^/CD8^+^ T cells in the tumors overexpressing C5a (88). However, more recently, anaphylatoxins generated by complement activation in the tumor microenvironment frequently associated with inhibition of anti-tumor immune responses important in metastatic spread. Pentraxin-related protein 3 (PTX3) has been identified as a tumor suppressor negatively regulating complement-mediated inflammation. PTX3 is shown to interact with C1q and Factor H to impede complement activation, resulting in lower C5a production, macrophage infiltration, and angiogenesis (89) (see Figure 2). In addition, Markiewski et al. showed that C5a aided in the recruitment of myeloid-derived suppressor cells (MDSCs) into tumors and inhibited anti-tumor T cell responses through the generation of reactive nitrogen and oxygen species (73). Consequently, blockage of C5a to C5aR signaling impaired tumor growth and lowered the percentage of MDSCs in spleens of lung cancer-bearing mice (90). C3a was also shown to be implicated with tumor progression. Namely, in a syngeneic primary murine B16-F0 melanoma model, the absence of C3aR signaling slowed tumor progression. In the same study, the authors showed that the antitumor effects of C3aR inhibition are linked with a decrease in tumor-associated macrophages and an increase in tumor-infiltrating neutrophils and CD4^+^ T lymphocytes (74). Moreover, anaphylatoxins are also shown to induce inflammation through the induction of bioactive molecules within the tumor microenvironment. For instance, C3a and C5a signaling enhances IL-6 production in astrocytoma cells (91), and blocking C5aR signaling down regulated the expression of IL-6 in a mouse model of lung carcinogenesis (90). Clinical studies have revealed that increased serum IL-6 in patients are associated with advanced tumor stages of various cancers, including multiple myeloma (92), non-small cell lung carcinoma (93), colorectal cancer (94), renal cell carcinoma (95, 96), breast cancer (97), and ovarian cancer (98). Taken all together, anaphylatoxins frequently hinder anti-tumorigenic immune responses and may be a potential target for therapeutics.

## Complement Inhibitors and Regulators

Complement activation is important for clearance of foreign agents, but pathogens have developed a number of strategies to evade the complement-mediated immune response. Most pathogens express soluble and surface-bound complement regulators that delay or even block complement effector functions in order to protect themselves from elimination. However, during a persistent infection, complement activation must be tightly regulated in order to protect host bystander cells. Therefore, it is not surprising that dysregulation of the complement cascade can result in autoimmune disease. Although the activation and deposition of complement products in tumor tissue has been demonstrated, the functional implication remains unclear.

Complement pathway participates in all facets of immune surveillance by collaborating with both the innate and adaptive immune systems. This “bridging” ability seems to continue as the activation products of C3 degrades into iC3b and C3dg. iC3b and C3dg are shown to bind to CR2 (CD21) on B cells (see Figure 2) to augment the immune response when limited amounts of antigen are available (99, 100). Furthermore, iC3b and C3dg aid in memory B cell induction and maintenance in the germinal centers and facilitates the shuttling of antigens between B cells and follicular dendritic cells by opsonizing cellular particles (66, 99, 101). In turn, the robust production of antibody against specific antigens improves the innate immune response by facilitating C1q-mediated activation of complement mentioned above.

Given the regulatory role of complement inhibitors on complement activation, it is tempting to hypothesize that cancer cells actively escape complement and immune surveillance by expressing complement inhibitors. The expression of membrane-bound inhibitors (CD46, CD55, and CD59) are upregulated among bladder cancer patients (102). It appears that different cancer types utilize different complement regulators to evade the complement mediated immune surveillance.

## Pathways of Complement Activation in Cancer

Although the mechanisms of complement activation in NSCLC are incompletely understood, pre-clinical and clinical data suggests that activation occurs at least in part through the classical pathway (72). IgM is a potent activator of the classical pathway, and we have observed deposits of IgM in experimental and human NSCLC (72). “Natural” IgM refers to germline encoded IgM that is produced even without exposure to specific antigen (103, 104). It is frequently poly-reactive, and there is evidence that natural antibodies bind to epitopes expressed on cancer cells (105). Although anaphylatoxins can suppress anti-tumor immunity, the MAC is directly cytotoxic. To protect themselves from MAC-mediated lysis, cancer cells express high levels of complement regulatory proteins, including CD46, CD55, and CD59 (64) (see Figure 2). Regulatory proteins CD46 and CD55 inhibit complement activation by binding with either C3b or C4b and preventing the formation of C3 and C5 convertases, while CD59 inhibits the MAC complex (82). We propose that complement is activated in the setting of cancer or precancer due to binding of natural IgM to neoantigens on the cell surface. This may lead to lysis of some target cells, but the tumor cells evolve mechanisms to evade complement-mediated elimination (such as overexpression of the regulatory proteins), and in fact employ byproducts of complement activation (anaphylatoxins) to suppress anti-tumor immunity in the TME. Thus, tumors can co-opt the immunosuppressive effects of complement activation while escaping its cytotoxic effects. Although IgM is primarily a classical pathway activator, in some instances mannose binding lectins bind to glycosylated IgM and activate the lectin pathway (106). Furthermore, even when complement is activated through the classical or lectin pathway, the alternative pathway amplifies the process and can account for the majority of overall activation (107). Thus, the complement system can be activated by many different protein-protein interactions, and activation generates multiple biologically active fragments. Drugs that selectively block activation through specific pathways are being developed and may be more effective and safer than the currently available drugs (108). Therefore, identification of the specific mechanisms of complement activation in NSCLC may lead to new treatment strategies for this disease. In addition, a more detailed examination of the role of individual regulatory proteins needs to be undertaken in preclinical models.

## Role of Cancer Cell Complement

**Figure 3:**
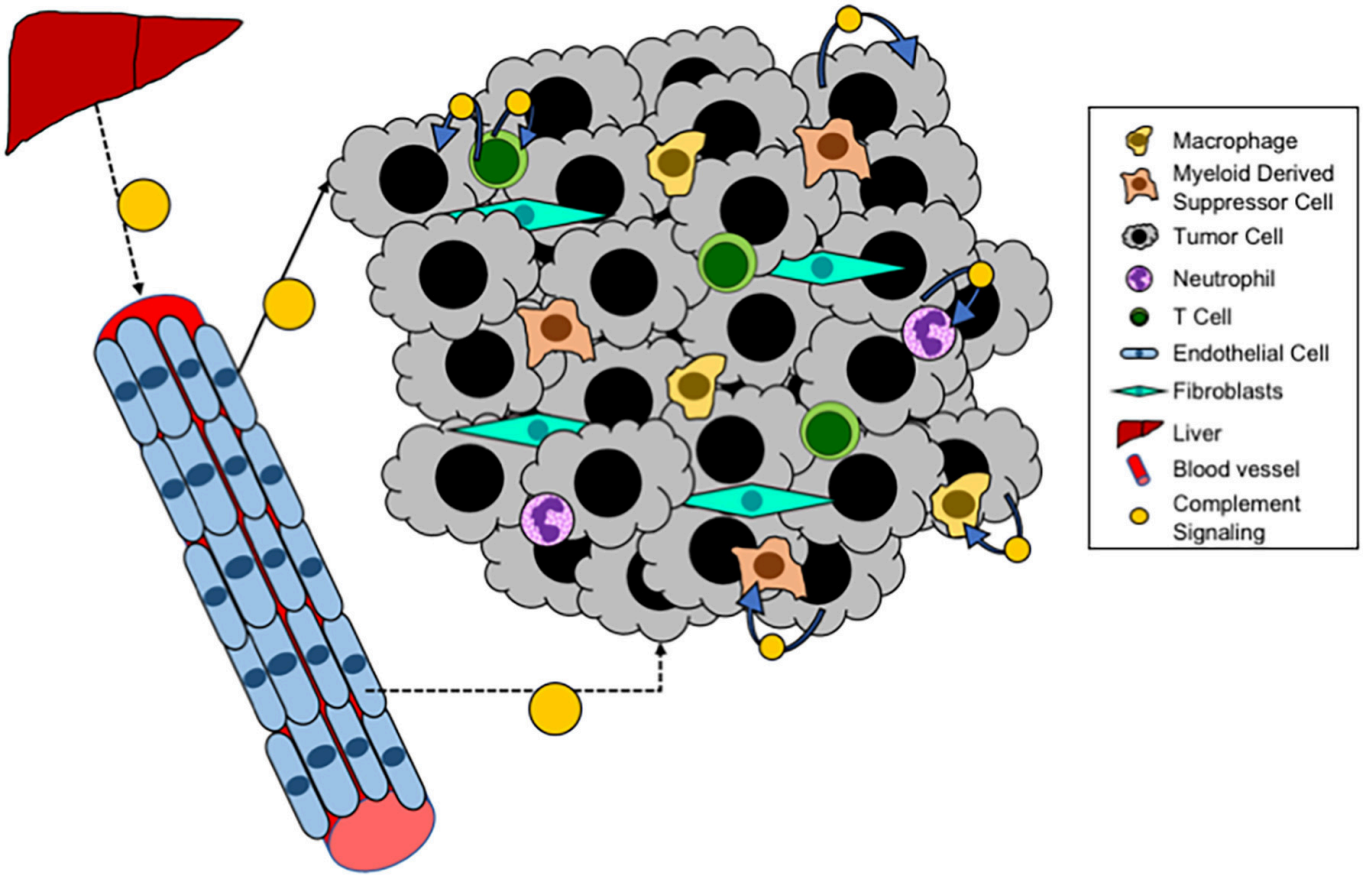
Local complement signaling in tumors. A schematic of complement signaling that occurs within tumors. Systemic complement is produced by the liver and travels via the blood to distant sites. While locally, within a tumor, tumor cells, T cells, and endothelial cells can all produce complement that acts either in an autocrine or paracrine fashion.

In addition to systemic complement activation, recent studies have demonstrated that cancer cells can also produce complement (see Figure 3). In ovarian cancer cells an autocrine loop in which expression of C3 by the cancer cells results in production of C3a which signals through the C3aR to promote growth (109). In this setting the role of cancer cell expression appears to be more critical for tumor progression than production by the TME, since these tumors grow equally well in C3^−/−^ mice as in WT. Overexpression of C5aR has been detected in both human lung cancer cell lines and in samples of human tumors (110). Elevated levels of expression have been associated with increased metastasis, and negatively with levels of E-cadherin, suggesting a role for C5a/C5aR signaling in regulating the epithelial-mesenchymal transition (EMT) of cancer cells. Consistent with these findings, in ovarian cancer expression of C3 is regulated by TWIST, which controls EMT (111). Data from our laboratory has demonstrated endogenous expression of C3 and production of C3a by Lewis Lung Carcinoma cells, which represent a mesenchymal phenotype (72). Recently, studies have demonstrated that intracellular activation of complement in cancer cells can act as an immunosuppressive pathway to regulate expression of PD-L1 (112). The pathways whereby cancer cell-intrinsic complement acts, as distinct to activation in the TME are likely to be different. In particular, cancer-cell intrinsic complement may signal in an autocrine fashion to promote cancer cell growth. However, this will be dependent not only on the complement activation, but also the expression of receptors for C3a and C5a on the cancer cells themselves.

## Role of Complement Production by Cells of the TME

Recent studies have demonstrated that T cells produce complement proteins which can act in an autocrine fashion to promote T cell function (see Figure 3). Studies have demonstrated that the promotion of a Th1 phenotype is promoted through translocation of C3a to the surface of CD4^+^ T cells, resulting in production of Th1 cytokines (113–115). Intracellular expression and function of C5 has also been shown (116). For both of these systems it appears that intracellular complement is critical for both the initiation and the contraction of T cell activation and IFNγ production. Thus, we anticipate that activation of intracellular complement would result in a greater proportion of anti-tumorigenic T cells (Th1), and thus blocking this pathway would be expected to promote rather than inhibit tumor progression. In other models, complement signaling has been shown to inhibit the function of Tregs, through pathways that involve both C3a and C5a (68). Since increased Treg infiltration of tumors is associated with immunosuppression, complement activation in this context would be predicted to inhibit tumor progression.

Complement activation has also been shown to occur in tumor endothelial cells (117). While the role of this pathway has not been extensively studied, data suggest that complement activation on endothelial cells allow for increased T cell homing and tumor infiltration. In this model activation of complement would appear to be critical for T cell infiltration associated with inhibition of tumor growth and increased sensitivity to immunotherapy, whereas complement inhibition would result in tumors with fewer T cells. Complement proteins are also expressed in other cells including macrophages and B cells (113, 118). Studies have shown production and activation in the setting of antigen presenting cells (APC) interacting with T cells, resulting in T cell activation. It would be expected that this activation would be associated with inhibition of tumor progression.

## Preclinical Models for Lung Cancer

To develop a better understanding of the role of complement in LUAD preclinical models that reproduce the human disease are required. Murine models for the study of lung cancer have been the backbone of preclinical data to support human clinical trials. Before focusing on the complement pathway, we will briefly discuss these models, and describe their strengths and limitations in defining immunoregulatory pathways. For more detailed information there are a number of excellent reviews on this topic (119, 120).

Early models examined tumor initiation in mice using carcinogens, including compounds present in cigarette smoke (119, 121). These mice develop lung adenocarcinomas with molecular, morphologic, and histologic similarities to that of human lung tumors (122). Both human and mouse adenocarcinomas arise from the type II epithelial cells or Clara cells of the peripheral lung and follow the same stages of development beginning with an initiated cell with a genetic mutation that proliferates to become a hyperplasia to a carcinoma *in situ*. Studying molecular and cellular mechanisms of murine lung tumor progression throughout the multi-stage carcinogenesis offers a better understanding of the pathogenesis. However, carcinogen-induced lung tumors are largely characterized by KRAS mutations, and there are currently no chemical models which result in tumors with other oncogenic drivers (123). Furthermore, these tumors are generally benign adenomas, which may eventually become invasive but do not metastasize. There are many carcinogenic agents available. Namely, most potent of all are the cigarette smoke carcinogens, such as polycyclic aromatic hydrocarbons (PAH), tobacco-specific nitrosamine, and benzo[a]pyrene (121). However, a 5-month exposure period with cigarette smoke carcinogens must be followed by a 4-month recovery period (124).

Genetic mouse models (GEMMs) with specific drivers have been developed. Using Cre-Lox technology lung tumors have been generated with Kras mutations (125, 126), and mutations in Egfr (24, 127). Using CRISPR technology, lung tumors driven by the fusion kinase Eml4-Alk have also been generated (128). By selectively deleting tumor suppressor genes such as p53, these tumors can be made to be more aggressive (129). A strength of this model is that, like the earlier chemical models, the various stages of tumor development can be recapitulated, and changes in the microenvironment can be assessed in a dynamic fashion. However, one significant limitation in this model is the low degree on non-synonymous mutations in the tumors (130). The mutational burden in these tumors is at least an order of magnitude less than seen in human LUAD. A consequence of this is the poor response to immunotherapy, such as checkpoint inhibitors. This is likely due to lack of neoantigens and recognition by adaptive immune cells (CD8^+^ and CD4^+^). In examining how pathways such as complement interact with the adaptive immune system, this may be a problem with many of these GEMMs.

Patient-derived xenograft (PDX) models require the injection of patient-derived cancer tissue into immunodeficient mice, such as Prkdc^scid^, Nude, or Rag1^null^ mice. The detailed differences among these strains will not be discussed here, but these immune-deficient mouse models all lack functional T cells and B cells. PDX offers a powerful tool to assess human tumor biology, namely the identification of therapeutic targets for the donating patients (131). In addition, many immunodeficient models accept allogeneic and xenogeneic grafts making them ideal models for cell transfer experiments or to examine tumor response to therapy *in vivo* prior to translation into clinical trials (131). One of the obvious advantages of PDX over implantation of human cell lines is that PDX represents more accurate tumor heterogeneity compared to the established human cell lines. Despite the advantages, xenograft models do not give insights to the role of the immune system in controlling tumor progression. Recent advances in immunotherapies stressed the importance of the immune system in tumor biology, and many strides have been made to create the next-generation PDX models with humanized mice. To establish PDX models conditioned with human immune system, CD34^+^ human hematopoietic stem cells (HSCs) are engrafted into host immunodeficient mice (132). HSCs give rise to various lineages of human blood cells in mice and to further improve the integrity of transplanted HSCs, immunocompromised mouse strains such as NOG-GM3, NSG-SGM3, and MISTRG are generated (133).

Implantable models involve the injection of cancer cells into mice. Earlier studies employed xenograft approaches where human NSCLC were injected subcutaneously into immunodeficient mice (134, 135). These studies allow a dynamic measurement of tumor growth, but suffer from the same limitations discussed above for PDX models. More recently, syngeneic models have been studied, in which immunologically compatible murine cancer cells are implanted into immunocompetent mice. The major disadvantage of this model is its limited number of cell lines in different mouse strains. For example, Lewis lung carcinoma (LLC) and CMT167 are the only Kras driven LUAD cell lines derived from the lung tumors of C57/BL6 mouse (136). Cell lines have been generated with Eml/Alk fusions derived from the CRISPR engineered mice (128), but there are currently to our knowledge no murine cell lines harboring Egfr mutations, and the current mouse models in which mutated Egfr is driven off a lung-specific promoter are unlikely to generate cell lines *ex vivo*, due to turning off of the promoter. Implantable models have the advantage of monitoring the progression of a full-fledged tumor, and in most cases these tumors will metastasize. Most murine cancer cells have high levels of non-synonymous mutations, and express neoantigens which are recognized by the adaptive immune system of the host. This system is also amenable to genetic manipulation of either the cancer cells, through silencing or overexpressing specific genes, as well as the host through the use of genetic knockout and targeted knockout mice. In using syngeneic implantable models, we would argue that it is critical to implant the tumors into the lung, rather than subcutaneously. This allows tumor development in the correct microenvironment. For example, tumors implanted subcutaneously will not be exposed to alveolar macrophages and other lung-specific cells. Despite its disadvantages, a syngeneic model—especially when combined with orthotopic injections—is the only currently available approach in which the tumor microenvironment is accurately depicted in the animal (72, 136–140).

Studies of the complement pathway in orthotopic immunocompetent models of LUAD have compared the effects of this pathway using a panel of murine lung cancer cell lines encompassing different oncogenic drivers (72). Both genetic and pharmacologic inhibition of complement blocked tumor progression, similar to what has previously been reported looking at metastasis to the lung (73, 75, 141).

## Clinical Targeting of Complement

Several studies have examined complement activation in human cancers, including lung cancer. Using a specific antibody against complement C4d, it has been shown that levels of this protein in plasma from lung cancer patients assessed by ELISA were able to discriminate between benign and malignant nodules (142). Published data also shows that the complement system is activated in many human patients with lung cancer (72, 76). Furthermore, examination of data in the Cancer Genome Atlas (TCGA) reveals gene amplification or increased expression of complement regulatory proteins, CD46 and CD55 in ~25% of human lung adenocarcinomas.

While preclinical data indicate that complement inhibitors may represent a novel therapeutic strategy for treating cancer in general and lung cancer in particular, there are currently no open clinical trials in any malignancy according to Clinicaltrials.gov. However, there is at least one FDA-approved complement inhibitor, ecoluzimab, which is a monoclonal antibody against C5. This agent has been approved for paraoxysmal nocturnal hemoglobinuria (PNH) (143). PNH is a hematological disorder where certain surface proteins are missing on erythrocytes (144). As related to the complement pathway, CD55 and CD59 expression is deficient in PNH (while CD46 is not normally expressed on human erythrocytes), thus preventing regulation of the complement cascade and leading to unregulated activation (144, 145). A number of other agents are being developed for other diseases (see Table 2). There are several important issues that need to be addressed to accelerate the application of complement inhibitors into the clinic. One of these is the choice of agent. Preclincal studies indicate that inhibition of either C3a or C5a signaling inhibit cancer progression in lung cancer models and in other malignancies. It is not clear if these signal through redundant pathways and thus the choice of agent needs to be more clearly developed. A second major issue is related to patient selection. In lung cancer, trials with unselected patients have in general been less successful than targeted trials with clear criteria for patient selection. As discussed above, the majority of currently ongoing clinical trials have focused on subsets of LUAD based on oncogenic drivers. In preclinical models, there is insufficient data to determine if complement inhibitors will be more effective for lung tumors with specific drivers, e.g., KRAS mutations vs. EGFR mutations vs. fusion kinases. This is complicated by the lack of appropriate models for many of these oncogenic drivers. Thus, additional preclinical studies will be needed to answer this. There is also a critical need to define biomarkers predictive of response to complement inhibitors. Since an important mechanism of complement inhibitors is modulating the immune system, it would be predicted that more immunogenic tumors with higher levels of infiltrating T cells would be more responsive to these agents. Thus, mutational burden might be predictive of response. However, in preclinical studies, EML4-ALK tumors, which have a relatively low mutational burden were shown to be sensitive to both C3aR or C5aR inhibition (72).

**Table 2:** Current drug candidates to target complement proteins.

**Target**	**Product (company)**	**Suggested indications**
C5aRA	PMX-53 (Peptech Ltd.)	RA, psoriasis
C5	Eculizumab/Soliris (Alexion Pharmaceuticals)	PNH
C5	Pexelizumab (Alexion Pharmaceuticals)	Clinical phase 3 for AMI, CABG
CD35 (CR1)	sCR1/TP10 (Avant Immunotherapeutics)	Clinical phase 2 for CABG
CD55 (DAF) and CD46 (MCP)	CAB-2/MLN-2222 (Millenium Pharmaceuticals)	Clinical Phase 1 for CABG
fH	fD inhibitor (Ra Pharma)	AMD, orphan renal diseases
C3	AMY-103 (Amyndas)	Transplant
C3	Compstatin/POT-4 (Potentia Pharmaceuticals)	Clinical phase 1 for AMD
C1-INH	Phucin/rhC1INH (Pharming Group N.V.)	Clinical phase 3 for HAE
C1r/C1s	C1-INH (Cetor, BerinertP, Leve Pharma)	Clinical phase 3 for HAE

*C5aRA, C5a receptor antagonist; RA, rheumatoid arthritis; PNH, paraoxysmal nocturnal hemoglobinuria; AMI, acute myocardial infraction; CABG, coronary artery bypass grafting; AMD, age-related macular degeneration; HAE, hereditary angioedema (146)*.

To date, there has been limited examination of complement activation using samples from human lung cancer. Early studies demonstrated that human NSCLC cell lines express high levels of complement inhibitory proteins and are resistant to complement-mediated lysis (147); these studies did not associate this with specific oncogenic drivers. Studies have used immunostaining of human lung tumor samples for expression of C3 and demonstrated association of expression with progression (148). These data suggest that complement activation in biopsies from cancer patients could represent a potential biomarker for local tumor activation of complement. Data from our laboratory has confirmed that complement activation as assessed by immunostaining for C3d represents a fairly frequent event in human lung cancer, with positive staining observed in approximately 40% of cases (72). However, additional studies are required to examine specific subgroups based on oncogenic drivers.

In KRAS driven lung cancer, response rates to anti-PD-1 therapy are approximately 20%. At least one study has demonstrated additivity of complement inhibitors targeting C5aR with anti-PD-1 in a mouse model where cancer cells are implanted subcutaneously (71). While these studies need to be extended to more clinically relevant models of lung cancer, they support a clinical trial using combinations of C5aR inhibitors and immune checkpoint inhibitors. Less is known regarding the effectiveness of complement inhibition for lung cancers with other drivers. Our laboratory has used a panel of murine lung cancer cells expressing the oncogenic fusion kinase Eml4-Alk. These cell lines were derived from a genetic mouse model employing a CRISPR construct to engineer the fusion kinase (128). Interestingly, there tumors appear to be resistant to anti-PD-1 therapy (72) similar to what is observed in clinical trials of patients with ALK fusion drivers. However, in an orthotopic mouse model these tumors were sensitive to inhibitors of either the C3aR or C5aR.

## Targeting Complement in Lung Cancer Prevention

While there is much active research focusing on treating established lung cancer, there is less known regarding the role of complement in the early stages of tumor initiation. Since lung cancer usually presents as advanced disease, it is appealing to develop strategies to prevent the initiation of lung tumors and/or inhibit the early stages of transformation. In that regard, there has been extensive efforts to develop chemopreventive agents for lung cancer (6, 149, 150). Preclinical studies have tested numerous agents preventing the development of lung tumors in mice. For example, recent studies have demonstrated that elevated levels of the lipid mediator prostacyclin can inhibit induction of lung tumors in response to either carcinogens or exposure to cigarette smoke (151, 152). This resulted in a clinical trial in which orally active prostacyclin analogs were able to inhibit progression of dysplastic lesions in smokers (153).

The role of the complement pathway has not been studied in any of these models. However, recent data suggest that complement activation is required for the formation of sarcomas (89). In these studies, chronic inflammation driven by the loss of PTX3 resulted in activation of the complement cascade which was critical for tumor formation. However, other studies have suggested that increased levels of Factor H are associated with increased risk of developing lung cancer (154, 155). Thus, additional studies are needed to define the precise role of the complement pathway in tumor initiation. These will require the appropriate mouse models, and a detailed examination of how differences in the oncogenes driving tumor initiation interact with the complement system.

## Concluding Remarks

In examining the findings regarding complement activation, it is clear that activation can result in pathways that can either promote or inhibit tumor progression. While anaphylatoxins can lead to engagement of immune-evading mechanisms, localized activation of intracellular complement in T cells can lead to production of CD4^+^ subpopulations which are associated with inhibition of tumor progression (113). Thus, there are competing pathways, and the net effect of inhibiting (or activating) complement in a particular cancer subtype such as lung cancer must be considered with great care. In addition, elevated expression of complement regulatory proteins have the potential to block the inhibitory effects of complement on cancer cell progression, while enabling the pro-tumorigenic and inflammatory effects (63, 156).

The efficacy of immunotherapy in multiple cancers including lung cancer support examining other pathways that regulate the immune response to tumors. Complement has emerged as a critical link between the innate and adaptive immune system, and therefore targeting this pathway as a therapeutic and preventative strategy in lung cancer has great potential. The complexity of complement has increased as our understanding has encompassed not just systemic complement activation, but also localized and intracellular complement regulation. From these studies it has become evident that complement can regulate both pro- and anti- tumorigenic pathways, and that activation in different cell types potentially will have opposing effects. This does not appear to be unique to the complement pathway; for example studies have shown in hepatocellular carcinoma that activation of NF-κB in hepatocytes vis a vis macrophages has opposing effects on tumor progression (157). Therefore, administration of therapeutic inhibitors (or activators) of the complement pathway may target competing, and potentially opposing pathways. Developing a strategy to selectively target the pro-tumorigenic effects of complement, and potentially simultaneously stimulate the anti-tumorigenic effects will require a deeper understanding of the role of these pathways in the tumor microenvironment. It is also likely that complement inhibitors will be used in combination with other therapies. For lung cancer these will likely be chemotherapy, targeted therapies, or immunotherapy. Currently, therapeutic strategies are dictated by the identification of specific oncogenic drivers. Therefore, examining complement in the context of these drivers in relevant preclinical models will be important in designing these trials.

Targeting the immune system as a therapeutic for cancer has revolutionized oncology. However, it is early days, and many potential targets regulating anti-tumor immunity have yet to be studied. Complement, as a bridge between innate and adaptive immunity is certain to be a potential target for treating lung cancer in the future.

## Author Contributions

All authors listed have made a substantial, direct and intellectual contribution to the work, and approved it for publication.

### Conflict of Interest Statement

The authors declare that the research was conducted in the absence of any commercial or financial relationships that could be construed as a potential conflict of interest.
